# How do energy price, energy-saving policies, and crisis affect energy-saving behavior?

**DOI:** 10.1016/j.heliyon.2025.e42787

**Published:** 2025-02-19

**Authors:** Dat Ngoc Nguyen, Duy Van Nguyen, Dat Dinh Nguyen, Thuy Trong Than, Loc Xuan Tran

**Affiliations:** a Foreign Trade University, Hanoi, Viet Nam; b Faculty of Economics and Business, Phenikaa University, Hanoi 12116, Viet Nam; c Ho Chi Minh City University of Industry and Trade, Ho Chi Minh, Viet Nam

**Keywords:** Green consumption, Energy-saving behavior, Household, Energy price, Energy-saving policies, Crisis, Vietnam

## Abstract

In order to reduce CO2 emissions into the environment, energy conservation is considered a long-term strategy. To encourage people to save energy, the government may need to implement certain policies. Vietnam is a developing country dependent on fossil fuels and heavily affected by climate change. At the same time, there has been no research evaluating the impact of energy prices, government policies, and crises on energy-saving behavior in Vietnam. Therefore, this study uses the theory of planned behavior, technological acceptance behavior, and social exchange to evaluate these relationships. This research will answer the keys research question such as how would the policies which related to price, energy-saving activities effect on energy-saving behavior of households; How would a crisis like covid-19 affect energy-saving behavior? The research conducted surveys and analysis on 1166 respondents. The results of the PLS-SEM model analysis indicate that energy-saving policies do not directly affect the intention to save energy but have a positive impact on energy-saving behavior. Both energy prices and crises have a positive impact on both the intention and behavior of energy-saving. The study has made a significant contribution to the theory by confirming the existence of social exchange theory and the protection motivation theory in explaining energy-saving behavior.

## 1 Introduction

Energy and electricity are considered a mandatory condition for national development. At the same time, with increasing development around the world, the demand for electricity and energy is increasingly higher [1,2]. The development of electricity holds significance for nations across various dimensions [3,4]. It serves as a crucial indicator of a country's economic progress, with nations exhibiting high electricity consumption often reflecting advanced levels of industrialization and economic development [5,6]. Electricity plays a pivotal role in powering industries, supporting services, facilitating transportation, and driving various economic activities [7]. In terms of social development, electricity consumption is vital for delivering essential services to the population, encompassing lighting, heating, air conditioning, and other household appliances [5,8]. Beyond enhancing people's quality of life and comfort, electricity creates conducive environments for educational, healthcare, and telecommunications initiatives. Furthermore, electricity consumption acts as a significant catalyst for infrastructure investment [9]. Increased electricity usage often coincides with the construction and enhancement of power plants, power grids, substations, and other infrastructure elements related to the power sector [9]. This facilitates the expansion and improvement of the power supply system to meet the growing needs of the nation. This brings challenges to the energy industry in particular and countries around the world in particular [1,2]. As a result, policies aimed at conserving energy are essential for decreasing energy consumption and improving the efficient utilization of available energy resources ([1,2]. In safeguarding the environment, bolstering energy security, and promoting sustainable development, it is imperative to prioritize this aspect within policies [10].

Achieving energy savings, whether in the industrial or consumer sectors, requires implementing conservation activities both at home and in manufacturing industries [10]. While demanding reduced energy consumption in manufacturing can be challenging for developing economies due to high investment costs and slow technology renewal, focusing on energy-saving behaviors in households becomes a more feasible approach [[10], [11], [12]]. Scholars worldwide have increasingly recognized the importance of energy-saving behavior in homes. Strategies to regulate and adjust individual-level saving behaviors have been proposed to reduce energy consumption successfully [13]. Research confirms that considering factors influencing energy-saving behavior at a personal level is vital in achieving effective energy conservation [1,2,14].

The pricing of electricity plays a crucial role in shaping and governing household electricity consumption behavior [[15], [16], [17]]. Comprehending the intricate relationship between electric prices and household electricity consumption behavior holds significant implications for devising policies, strategies, and measures to encourage energy conservation and efficient utilization [[15], [16], [17]]. Firstly, electric prices exert influence on the selection and utilization of energy-efficient household appliances [[15], [16], [17]]. With an increase in electric prices, consumers tend to invest in more energy-efficient appliances as a means to curtail electricity consumption and associated costs [[18], [19], [20]]. This shift is evident in the widespread adoption of LED lamps over traditional incandescent ones, and the preference for refrigerators and air conditioners with higher electric efficiency indices [[15], [16], [17]]. These choices contribute to energy savings, enhance overall efficiency, and mitigate greenhouse gas emissions [21]. Secondly, variations in energy prices impact individuals' usage patterns of electrical appliances [[15], [16], [17]]. As electric prices escalate, consumers often contemplate altering their consumption habits to reduce electricity usage and cut costs [22]. This may involve reducing the duration of appliance usage, turning off lights when not needed, optimizing the operation of washing machines and dishwashers during periods of low demand, and adjusting the temperature settings of air conditioners and heaters accordingly [22,23]. These modifications not only result in reduced electricity usage but also cultivate an ethos of energy preservation within the community, thereby aiding in the establishment of a more sustainable society [22,23].

Certainly, scholars have delved into the repercussions of crises on both income levels, and the energy-saving practices of the general population [[24], [25], [26]]. Notably, there has been a substantial focus on the impact of the COVID-19 pandemic from 2020 onwards (Considering COVID-19 as a crisis). Apart from presenting health hazards, the pandemic has heightened the susceptibility to job losses and diminished income during unpredictable periods [27]. Consequently, following the protective motivation theory, the risks associated with employment may drive individuals to adopt economically safeguarding behaviors, including the adoption of energy-saving and efficient measures.

Due to the importance of energy-saving activities, research on energy-saving behavior has also been noticed by many scholars worldwide [[24], [25], [26]]. Studies have often focused on the effect of perception on saving intention and behavior [23,28]. These studies assume that when individuals are more aware of the importance of energy/electricity saving activities at the individual level, they are more willing to practice saving behaviors [23]. Furthermore, when individuals have knowledge, concern, and responsibility for saving electricity and saving energy, they will put more effort into implementing saving behaviors [23]. Some other studies have examined the influence of context on saving behavior [15]. These studies assume that external stimuli and pressures can induce changes in individuals' energy-saving behaviors. For example, government policies encouraging people to use energy-saving appliances can also influence user behaviors [15]. Social norms about right behaviors are also a pressure to change individuals' energy-saving behavior [14,29].

In Vietnam, despite the government having implemented numerous policies related to energy-saving and efficiency, there has been no research measuring the effectiveness of these policies or evaluating their impact on the energy consumption behavior of the population. Therefore, this study aims to assess the impact of the state's energy-saving policies on the intention and behavior of households towards energy conservation. Previous studies have primarily explained the intention and behavior of energy conservation based on theoretical models such as TAM (Technology Acceptance Model) and TPB (Theory of Planned Behavior). This study applies the Social Exchange Theory (SET) to examine the effectiveness of the state's energy-saving policy and use the Protective Motivation Theory (PMT) to assess the impact of crisis and energy prices on the intention and behavior of energy conservation among individuals. This research will answer key research questions such as: How do policies related to price and energy-saving activities affect the energy-saving behavior of households? How does a crisis like COVID-19 impact energy-saving behavior? Overall, this study contributes to the literature by demonstrating that SET and PMT can explain the relationship between energy prices, energy-saving policies, crises, and household energy-saving behavior. Additionally, policymakers can use these insights to develop appropriate policies that enhance energy-saving behavior.

## 2 Literature review

### 2.1 The theories of energy-saving behavior

#### 2.1.1 Theory of planned behavior

The TPB is commonly used to study behavioral intentions. According to TPB, behavior is influenced by the intention to perform that behavior, determined by attitude, subjective norms, and perceived behavioral control [30,31] Attitude reflects an individual's positive or negative evaluation of a behavior, with a positive attitude increasing the likelihood of engagement and a negative attitude decreasing it [31,32]. Subjective norms refer to an individual's perception of social pressure from important others to perform or not perform a behavior, impacting compliance. Perceived behavioral control relates to an individual's perception of the ease or difficulty of performing a behavior, affecting their intention to perform it. However, in researching energy-saving behavior, policy factors and changes in energy knowledge also play a role important [15,33,34]. However, the studies use Therefore, this study incorporates characteristic factors like information policy and energy knowledge alongside TPB to analyze the intention to save energy behavior.

#### 2.1.2 The theory of technology acceptant model

The TAM examines external factors that influence consumers' attitudes toward technology use. The trust factors in TAM determine users' attitudes, influencing their behavioral intentions, with perceived usefulness directly impacting their perception of significance. Perceived usefulness is the belief that a system can improve performance [35]. Ease of use measures the level of comfort or discomfort in using a product. For energy-saving appliances with new technology, some consumers may perceive them as difficult to use and be reluctant to adopt them [36]. However, perceived ease of use positively affects perceived usefulness, where simplicity and convenience in operating a system enhance the perceived benefits of using energy-efficient appliances [36,37].

#### 2.1.3 The social exchange theory

The SET provides a compelling framework for understanding energy-saving behaviors by emphasizing the reciprocal nature of interactions between individuals and their social environments. According to SET, individuals are motivated to engage in energy-saving actions when they perceive a balance between the costs and benefits of such behaviors, often influenced by social norms and expectations [31,38,39]. Research indicates that high levels of social capital, characterized by trust and mutual support within communities, significantly enhance pro-environmental behaviors, suggesting that individuals are more likely to conserve energy when they feel a sense of obligation to their peers [38,39]. When households switch to energy-saving devices, they incur a conversion cost, so they would be more proactive if they received support from the government [39]. Additionally, the integration of social identity and SET highlights how individuals’ identification with pro-environmental groups can foster a sense of responsibility and commitment to energy-saving initiatives [40,41]. Thus, understanding the dynamics of social exchange can inform strategies aimed at promoting energy conservation through community engagement and social development.

#### 2.1.4 The protective motivation theory

The Protective motivation theory (PMT) offers a valuable framework for understanding energy-saving behaviors by emphasizing the cognitive processes that influence individuals' intentions to engage in pro-environmental actions. PMT posits that individuals assess threats and their coping abilities, which subsequently shapes their behavioral intentions. Research indicates that attitudes towards energy-saving, alongside cultural values, significantly influence these intentions, as demonstrated in studies that integrate the TPB with PMT [8]. Furthermore, normative factors, such as personal and social norms, have been shown to enhance the motivation for energy conservation, suggesting that individuals are more likely to adopt energy-saving behaviors when they perceive such actions as socially endorsed [[42], [43], [44]]. Additionally, the interplay between awareness and behavioral intention highlights the necessity of fostering a supportive environment that encourages energy-saving practices, particularly among urban residents [45].

### 2.2 Hypotheses

The study uses SET to explain the impact of energy-saving policy factors and people's intentions and behaviors. The SET is a social psychological concept that describes how individuals make decisions and form relationships based on the principles of cost and reward [46]. Energy-saving policies affect behavior through economic incentives, guiding residents to save energy [[47], [48], [49]]. These policies can change the economic costs of residents' energy consumption by setting energy prices, collecting energy use taxes, and subsidizing energy-efficient products. In addition to supporting policies to reduce energy costs with energy-saving devices, this policy also affects the perception of the need to save energy. Therefore, the research hypothesis is as follows:

H1a Energy policy has a positive effect on intention

H1b Energy policy has a positive impact on energy-saving behavior

PMT is also used in this study to explain the relationship between energy prices and energy-saving intentions and behaviors. The defensive motivation theory was developed by Roger [50]. PMT indicates self-protection against external threats. This study considered energy prices and COVID-19 as two income-related (economic) threats to households [46]. The relevant literature on the relationship between energy prices and energy consumption shows that increases in energy prices, taxes, and subsidies are positively related to energy savings [51,52]. But there are also a few studies that have shown that such economic policies are not necessarily effective and that their effects are different for households with varying levels of income or regions, and that financial measures have other consequences for groups with varying levels of price sensitivity [23,53,54]. PMT in this case, indicates when energy prices are assessed to be high and increase the risk of financial risk on bills will make people reduce energy consumption to protect the family economy. Therefore, the research hypothesis is put forward as follows:

H2a Energy price has a positive effect on intention

H2b Energy price has a positive impact on energy-saving behavior

The study considers Covid-19 as a crisis factor, resembling environmental, economic, or social fluctuations with significant impacts on people's lives and health [55,56]. The pandemic has altered residents' living situations, leading to concerns about income reduction and job loss. As per the PMT theory, Covid-19 heightens worries about expenditures, prompting individuals to consume less and use energy more efficiently to protect their family's economy. The research aims to explore the relationship between Covid-19 and energy-saving behavior, providing valuable insights for predicting responses to future crises. During the pandemic, Vietnam experienced changes in energy consumption patterns, with increased energy use due to social distancing measures. The hypothesis of the study is outlined below:

H3a Crisis has a positive effect on intention

H3b Crisis has a positive impact on behavior

Perceived behavioral control refers to the difficulty individuals perceive in carrying out energy-saving actions and the influence of external factors on their ability to do so. It is influenced by beliefs about their control and the resources available to them. Social roles, pressures, and ethical standards acquired through learning and imitation also impact behavior. These external factors, alongside personal beliefs and resources, play a significant role in shaping individual choices regarding energy-saving behaviors [43,57]. The concept is rooted in the TPB and acknowledges the interplay between personal agency and societal influences in shaping energy-saving actions [58,59]. Social incentives can encourage people to follow social norms and engage in greener consumption behavior. Social norms have a significant impact on childish behavior [60,61].

H4 Perceived behavioral control has a positive effect on intention

The subjective norm factor, as outlined in the TPB and supported by various literature studies, demonstrates a significant influence on both the intention to engage in energy-saving behavior and the subsequent actual behavior [62]. The personal criteria influencing behavioral subjects primarily stem from perceived social norms and the behavior exhibited by their reference group [43]. This study subjectively defines energy-saving behavior as the individual's performance in a given role, influenced by the energy-saving practices observed within their reference group, thus shaping their psychological perception [63]. This factor is drawn from the TPB.

H5 Subjective norm has a positive effect on intention

Attitude refers to the subjective evaluation of residents' specific behaviors related to energy-saving usage, formed by a blend of their beliefs about the behavior and their assessment of the outcomes associated with it [58,64] Behavioral beliefs are influential in determining whether an individual will partake in a specific activity, while outcome evaluation assesses the positive or negative consequences of that behavior [43,57]. Consequently, a more favorable attitude towards energy-saving appliances correlates with a greater inclination to pursue them. This factor is derived from the TPB.

H6 Attitude has a positive effect on the intention

Davis [65] proposed the TAM, which explains and predicts an individual's acceptance of new technology products. The TAM comprises two fundamental variables: perceived usefulness and perceived ease of use, which impact consumers' motivation and perceptions regarding the adoption of new technology products. Perceived usefulness pertains to the degree to which individuals perceive a given technology as advantageous to them [66]. Perceived ease of use, on the other hand, relates to the extent to which an individual believes that utilizing the new technology is straightforward. Some studies have shown that both perceived usefulness and perceived ease of use need to be examined from various perspectives [67,68]. Furthermore, scholars have suggested that incorporating relevant variables into the TAM model can enhance its explanatory power in a specific context [69]. The two factors, perceived usefulness and perceived ease of use, are derived from the TAM model of technology acceptance.

H7a Perceived ease of use has a positive effect on usefulness

H7b Perceived ease of use has a positive impact on energy-saving intention

H8a Perceived usefulness has a positive effect on attitudes about energy saving

H8b Perceived usefulness has a positive effect on energy-saving intention

H9 Energy-saving intention has a positive impact on energy-saving behavior

**Fig. 1:**
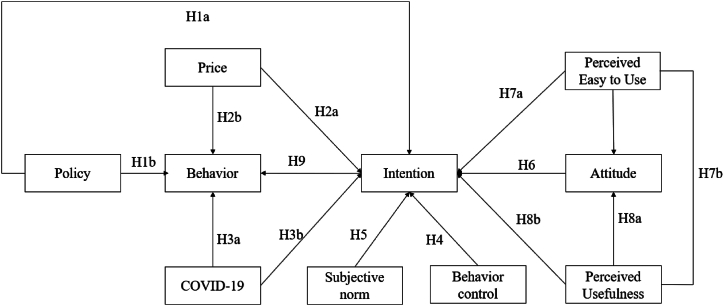
Research model.

The research model and hypotheses are presented in Fig. 1.

## 3 Methodology

### 3.1 Research design

This study used a structured questionnaire to collect data and test the proposed hypotheses. The constructs measured in the model were adapted from prior studies, utilizing existing items as a basis for assessment [15,23,27,55,70]. All constructs are considered first-order constructs. The questionnaire items were translated from English to Vietnamese and subjected to back-translation to maintain consistency in meaning throughout the translation process. The questionnaire, adapted from previous research, is provided in Table 2. Responses will be recorded using a 5 point Likert scale: 1- strongly disagree, and 5- strongly agree. The detailed in Table 2.

### 3.2 Sample

The study focused on households in various provinces and cities across Vietnam as its target population. Sample study was collected through two phases (1) preliminary data, and (2) official data. In the first step, the authors conducted a pilot survey to adjust scales and preliminary evaluation of reliability of constructs. In this step, the survey with 60 households in Ha Noi (North VietNam), Thanh Hoa (Middle VietNam) and Ho Chi Minh (South VietNam) is spread. The reliability test shown that Cronbach's Alpha of constructs all greater than 0.7 indicating the constructs in the model achieved reliability. After that, in the second step, the research used an official survey to collect data and test hypotheses in the research model. Data collection took place from October 2021 to May 2022, utilizing social networking platforms such as Facebook and email as primary channels for gathering information. Survey questionnaires were distributed to individuals who are either married (husband or wife) or single, currently working in the following provinces and cities: Hanoi (Northern Vietnam), Thanh Hoa (Central Vietnam), and Ho Chi Minh City (Southern Vietnam). The data collection was evenly distributed across the three regions of Vietnam. According to [NO_PRINTED_FORM] [71], a minimum of 100 samples is required for factor analysis. According to [NO_PRINTED_FORM] [72] for sample numbers below 100 is bad, 200 is good, 300 is good, 500 is very good, 1000 or more is excellent. With questionnaires distributed, data got 1166 valid respondents. The sample size reach excellent level for quantitative research [73,74]. This study was initiated under Degree No. 052022/QD-QAglobal issued by the Quantitative Analysis Center, QAglobal, Vietnam. All respondent's opinions are helpful in the research, and none of them are considered right or wrong. Personal information (if any) is kept confidential. They agree to participate in this study and respondents participated in the survey on a voluntary basis.

### 3.3 Data analysis

Data analysis was conducted using confirmatory factor analysis (CFA) with a sample size of 1216. The measurement model underwent tests for reliability and validity, ensuring composite reliabilities (CR) exceeding 0.5 and average variance extracted (AVE) surpassing 50 %. Additionally, convergence validity was assessed by ensuring factor loadings exceeded 0.5. Subsequently, discriminant validity was examined, where the square roots of AVE values should exceed the correlation coefficients between variables. Finally, research hypotheses were tested using Partial Least Squares Structural Equation Modeling (PLS-SEM), a widely used method in psychological research [75,76] and social science [77]. Especially, this model has been applied by many researchers in recent years to consider the influence of factors on energy-saving behavior [1,2].

## 4 Result

### 4.1 Descriptive

This study gathered data from 1166 valid participants. The information in Table 1.

**Table 1:** Demographic information of respondents (*n* = 1166).

	n = 1166	%
*Gender*		
Female	595	51.0
Male	571	49.0
*Education*		
High School	95	8.1
Colleage	76	6.5
Graduate University	828	71.0
Master/Phd.	167	14.3
*Occupation*		
Offical staff	584	50.1
Unemployment	15	1.3
Worker	70	6.0
Engineer	80	6.9
Self-employed	319	27.4
Lecturer/teacher	30	2.6
Others	68	5.8
*Income*		
under 10 million VND/month	54	4.6
10- under 15 million VND/month	184	15.8
15- under 20 million VND/month	415	35.6
>20 million VND/month	513	44.0

**Table 2:** Scales’ evaluation.

Constructs		Loading	Cronbach's Alpha	Composite Reliability	Average Variance Extracted (AVE)	Adapted
ATT	ATT1	0.664	0.708	0.834	0.630	Ru et al. [78]
	ATT2	0.872				
	ATT3	0.830				
BE	BE1	0.750	0.753	0.843	0.575	Zhang et al. [23]
	BE2	0.812				
	BE3	0.673				
	BE4	0.791				
CON	CON1	0.834	0.762	0.863	0.678	Fu et al. [15]
	CON2	0.821				
	CON3	0.814				
COVID	COVID1	0.834	0.774	0.869	0.689	Vo-Thanh et al. (2021)
	COVID2	0.784				
	COVID3	0.870				
INT	INT1	0.805	0.783	0.874	0.698	Zhang et al. [23]
	INT2	0.848				
	INT3	0.853				
PEU	PEU1	0.831	0.674	0.858	0.751	Zhang et al. [23]
	PEU2	0.901				
PO	PO1	0.924	0.811	0.914	0.841	Zhang et al. [23]
	PO2	0.910				
PRI	PRI1	0.922	0.919	0.949	0.86	Fu et al. [15]
	PRI2	0.940				
	PRI3	0.920				
PU	PU1	0.774	0.728	0.847	0.649	Ru et al. [78]
	PU2	0.848				
	PU3	0.792				
SNO	SNO1	0.811	0.806	0.886	0.721	Wang et al. [70]
	SNO2	0.853				
	SNO3	0.882				

### 4.2 Reliability test

The reliability assessment in PLS-SEM goes beyond just relying on Cronbach's Alpha and the composite reliability (CR). PLS-SEM also evaluates reliability using the factor loadings and the average variance extracted (AVE). A commonly accepted criterion for reliability is a composite reliability (CR) value greater than 0.7. The results of the analysis presented in Table 2 show that the reliability coefficients of all factors are greater than 0.7. Therefore, all the factors considered in the model achieve satisfactory reliability. To evaluate the convergence value of the scales, analysis on PLS-SEM analysis was conducted. The coefficients of factor loading greater than 0.5 and mean explanatory variance (AVE) greater than 0.5 were tested. The test results show that the factor loading coefficients of all observed variables are greater than 0.5 and the explanatory variance AVE of all factors is greater than 0.5. Therefore, all the factors considered in the model achieve convergent validity. The detail in Table 2.

### 4.3 Discriminant validity test

The study assessed discriminant validity following the procedure recommended by [NO_PRINTED_FORM] [79]. Table 3 illustrates that, except for the formative constructs where calculating AVE was not applicable, the square roots of the AVE values for the two reflective constructs (ranging between 0.758 and 0.927) substantially exceeded most of the corresponding bootstrapped correlation coefficients, which ranged from 0.421 to 0.719. This finding indicates a high level of discriminant validity.

**Table 3:** Discriminant validity.

	ATT	BE	CON	COVID	INT	PEU	PO	PRI	PU	SNO	
ATT	**0.794**										
BE	0.547	**0.758**									
CON	0.644	0.578	**0.823**								
COVID	0.579	0.595	0.632	**0.830**							
INT	0.580	0.665	0.599	0.645	**0.835**						
PEU	0.428	0.504	0.446	0.473	0.504	**0.867**					
PO	0.522	0.587	0.520	0.528	0.544	0.510	**0.917**				
PRI	0.488	0.718	0.507	0.510	0.621	0.421	0.479	**0.927**			
PU	0.503	0.682	0.587	0.631	0.677	0.497	0.578	0.572	**0.805**		
SNO	0.595	0.642	0.656	0.719	0.713	0.502	0.592	0.557	0.622	**0.849**	

*Notes*: 1st value = Correlation between variables (2-tailed *t*-test); Square root of AVE (bold diagonal).

### 4.4 Hypotheses testing

The results of the hypothesis testing using PLS-SEM (Partial Least Squares Structural Equation Modeling) show that the factor PO does not have a significant impact on INT (Intention) as it is insignificant at the 10 % level of significance. However, it is observed that PO does have a positive and significant impact on BE (β = 0.184 and significant at 5 %). PRI has a positive impact on INT (β = 0.193 and significant at 5 %). PRI has a positive impact on BE (β = 0.416 and significant at 5 %). COVID-19 crisis has a positive effect on INT (β = 0.084 and significant at 5 %). COVID-19 crisis has a positive effect on BE (β = 0.108 and significant at 5 %). SNO has a positive impact on INT (β = 0.287 and significant at 5 %). ATT has a positive impact on INT (β = 0.104 and significant at 5 %). PEU has a positive impact on PU (β = 0.497 and significant at 5 %). PEU has a positive impact on ATT (β = 0.236 and significant at 5 %). PU has a positive impact on ATT (β = 0.386 and significant at 5 %). PU has a positive impact on INT (β = 0.235 and significant at 5 %). The detail in Table 4.

**Table 4:** The PLS-SEM result.

	Beta	SE	T-Statistics	P Values	Hypotheses	Conclusion
ATT - > INT	0.104	0.028	3.703	0.000	H6	Accepted
**CON - > INT**	**0.025**	**0.031**	**0.821**	**0.412**	H4	**Rejected**
COVID - > BE	0.108	0.028	3.857	0.000	H3b	Accepted
COVID - > INT	0.084	0.028	2.980	0.003	H3a	Accepted
INT - > BE	0.178	0.036	5.001	0.000	H9	Accepted
PEU - > ATT	0.236	0.029	8.141	0.000	H7b	Accepted
PEU - > PU	0.497	0.030	16.559	0.000	H7a	Accepted
PO - > BE	0.184	0.025	7.310	0.000	H1b	Accepted
**PO - > INT**	**0.001**	**0.028**	**0.037**	**0.971**	H1a	**Rejected**
PRI - > BE	0.416	0.040	10.278	0.000	H2b	Accepted
PRI - > INT	0.193	0.025	7.633	0.000	H2a	Accepted
PU - > ATT	0.386	0.028	13.679	0.000	H8a	Accepted
PU - > INT	0.235	0.033	7.137	0.000	H8b	Accepted
SNO - > INT	0.287	0.032	9.090	0.000	H5	Accepted

*Control variable*						
gender - > BE	−0.037	0.018	2.113	0.035		
gender - > INT	−0.012	0.018	0.639	0.523		

Notes: PO: Policy; PRI: Price; COVID: COVID-19 crisis; CON: Control behavior; SNO: Subjective norm; ATT: Attitude; PEU: Perceived easy to use; PU: Perceived usefulness; INT is Intention; BE is Saving Behavior; numbers in brackets: standard error; ^a^: significance at 1 % respectively; ^b^: significance at 5 % respectively; ^c^: significance at 10 % respectively (two-tailed *t*-test).

In addition, we analysis the factor's role in research model. The results show that PU has mediating role between the relationship PEU-ATT. ATT and INT have mediating role between PEU-BE and the relationship PU-BE; INT has mediating role between the relationship ATT-BE; PU, ATT, and INT have mediating role between the relationship PEU-BE; INT has mediating role between the relationship COVID19-BE; INT has not mediating role between the relationship PO-BE; INT has mediating role between the relationship PRI-BE, PU-BE; SNO-BE; PU and INT have mediating role between the relationship PEU-BE; ATT has mediating role between the relationship PEU-INT, and PU-INT; PU has mediating role between the relationship PEU-INT; PU and ATT have mediating role between the relationship PEU-INT. The detail in Table 5.

**Table 5:** Analysing the mediating role of variables.

	Beta	Se	T-stats	P-Values
PEU - > PU - > ATT	0.192	0.020	9.832	0.000
PEU - > ATT - > INT - > BE	0.004	0.001	2.926	0.004
ATT - > INT - > BE	0.019	0.006	3.157	0.002
PU - > ATT - > INT - > BE	0.007	0.002	2.957	0.003
PEU - > PU - > ATT - > INT - > BE	0.004	0.001	2.879	0.004
**CON - > INT - > BE**	**0.004**	**0.006**	**0.770**	**0.442**
COVID - > INT - > BE	0.015	0.006	2.415	0.016
PEU - > INT - > BE	0.012	0.005	2.292	0.022
**PO - > INT - > BE**	**0.000**	**0.005**	**0.038**	**0.970**
PRI - > INT - > BE	0.034	0.007	4.691	0.000
PU - > INT - > BE	0.042	0.011	3.851	0.000
PEU - > PU - > INT - > BE	0.021	0.006	3.640	0.000
SNO - > INT - > BE	0.051	0.012	4.152	0.000
PEU - > ATT - > INT	0.025	0.007	3.502	0.001
PU - > ATT - > INT	0.040	0.011	3.546	0.000
PEU - > PU - > ATT - > INT	0.020	0.006	3.468	0.001
PEU - > PU - > INT	0.117	0.017	6.863	0.000

This result shows that PU, ATT, INT not only have a direct impact on household energy-saving behavior but also have a mediating role in the relationship in the research model. It can be seen that the results of direct and indirect impacts are similar. PO do not directly affect INT and also do not indirectly affect BE through INT. Therefore, this result shows that incentive policies seem to be insignificant to the intention to energy-saving. Mandatory policies may be useful when they will immediately change the energy saving behavior of households.

### 4.5 Discussion

Government energy-saving policies significantly influence energy-saving behaviors, primarily through financial incentives and increased public awareness. These policies create an environment conducive to energy-saving actions. For instance, subsidies for energy-efficient devices encourage households to adopt energy-saving practices [80,81]. Additionally, when individuals are informed about the economic benefits of energy-saving measures, they are more likely to act to take advantage of these benefits [80]. The TPB further elucidates this relationship, suggesting that while subjective norms influence intentions, actual behaviors are more directly affected by external factors like government policies [43,82]. Research indicates that moral obligation and awareness of energy-saving policies can enhance individuals' engagement in energy-saving behaviors [42]. Thus, the effectiveness of government policies lies in their ability to foster favorable conditions for energy-saving actions rather than merely influencing intentions. Overall, a comprehensive approach that combines financial incentives with effective communication of policy benefits is essential for promoting energy-saving behaviors among individuals [26,47,49].

PRI has a direct and positive impact on household's energy-saving behavior. The study findings suggest that when residents perceive high energy prices, they tend to increase energy-saving behaviors. When energy prices exceed the average income level of the population, individuals become more concerned about reducing household living costs. Specifically, reducing energy bills/electricity bills is one of the ways to cut household expenses and alleviate economic pressure. As energy prices rise, it often leads to an increase in the prices of essential goods, including food, which further escalates living costs. Therefore, residents not only limit their expenditures but also adopt more energy-saving behaviors. Moreover, some households are willing to invest in energy-saving products when energy prices increase. They recognize that investing in energy-saving appliances and products will bring economic efficiency in the short and long term. These investments are perceived as economically beneficial. The research findings are consistent with the results of previous studies conducted by other researchs [23,54,83].

The COVID-19 pandemic has catalyzed a significant shift in energy-saving behaviors among individuals and institutions. Research indicates that heightened awareness of energy consumption during the pandemic has led to more energy-efficient practices, as consumers became more conscious of their usage patterns [84]. The lockdown measures resulted in reduced energy consumption, particularly in educational institutions, where the transition to online learning decreased overall energy demand [85]. Psychological factors, such as increased risk perception and economic uncertainty, have also influenced energy-saving behaviors, prompting households to adopt more conservative energy practices [86]. Furthermore, normative influences, including personal and societal values, have been shown to correlate with energy-saving intentions and behaviors, reinforcing the importance of community norms in promoting sustainability [45]. Overall, the pandemic has not only altered immediate energy consumption patterns but also fostered a long-term shift towards more sustainable energy practices, suggesting that the crisis may have lasting implications for energy conservation efforts [87].

The SNO factor has a positive and significant impact on the intention to save energy and indirectly influences energy-saving behavior. The research findings suggest that social norms have a considerable influence on households' intentions to save energy, aligning with the TPB. When individuals live in an environment where energy-saving is a recognized norm, they are more likely to be influenced by that environment. This aligns with some studies that suggest subjective norms as a driving force for energy-saving behaviors, with long-term effects and explaining the willingness to engage in energy-saving actions [51,64]. In the Vietnamese context, culture and lifestyle play significant roles in shaping people's behavior, and social norms have a considerable impact. Vietnamese society places importance on collective values and the influence of group members on individual decisions. Therefore, when individuals perceive energy-saving as a socially accepted behavior, they are more likely to adopt such practices to fit into the social norms of their community.

Attitude has a positive and significant impact on the intention to save energy and indirectly influences energy-saving behavior. This finding is in line with the results of several previous studies, where attitude has been shown to influence both intention and behavior [23,[88], [89], [90]]. In the context of energy-saving behavior, individuals with positive attitudes towards the environment are more likely to exhibit energy-saving behaviors [91]. The results of this study highlight the crucial role of attitude in fostering the acceptance and adoption of energy-efficient products and practices within households. A positive attitude towards energy-saving may result from an individual's concern for environmental sustainability, cost-saving motivations, or awareness of the benefits of energy conservation. These positive attitudes can serve as strong drivers for the intention to save energy and, ultimately, lead to actual energy-saving actions. Understanding the influence of attitudes on energy-saving intentions and behaviors can aid policymakers and energy conservation advocates in designing targeted interventions and communication strategies. By promoting positive attitudes towards energy-saving, individuals are more likely to embrace energy-efficient technologies and adopt energy-saving practices in their daily lives, contributing to a more sustainable and environmentally friendly society.

PEU is an important factor in explaining why people use energy-saving products. In another study, it was also mentioned that PEU is a strong factor influencing the intention to adopt technology [92]. Thus, numerous studies and pieces of evidence have consistently demonstrated that the perceived ease of use of energy-saving products is a predictive factor for the intention to save energy. Perceived ease of use refers to individuals' perceptions of how effortless and straightforward it is to use a particular product or technology. In the context of energy-saving products, if individuals perceive these products as easy to understand and use, they are more likely to adopt them in their daily lives. The ease of use is a crucial factor as it can remove barriers that might hinder individuals from adopting energy-saving practices. When energy-saving products are perceived as user-friendly and convenient, individuals are more likely to feel confident in their ability to use them effectively. This positive perception can, in turn, lead to a higher intention to adopt energy-saving practices and contribute to energy conservation efforts.

The perceived usefulness of energy-saving products is considered to have a direct and positive impact on the intention to save energy and an indirect effect on energy-saving behavior of individuals. This result aligns with the findings of previous studies [[92], [93], [94]]. Perceived usefulness of energy-saving products refers to an individual's belief that a specific system will provide good performance and regular cost savings. The perception of ease of use also positively influences the perception of usefulness. This means that the difficulty of operating a new device can change people's perceptions of its usefulness because the more challenging the device is to use, the more time and effort it will require, thus reducing the perception of its usefulness. In this context, perceived usefulness is related to the visible benefits of using energy-saving devices. These devices are designed to reduce energy consumption in households, thus saving energy and protecting the environment. Therefore, it is hypothesized that consumers will be interested in energy-saving devices because they are perceived as useful in cutting energy bills and improving their quality of life.

## 5 Conclusion and implications

### 5.1 Conclusion

The study has systematically examined various theoretical models related to energy-saving behavior in households in Vietnam. The analysis was conducted on 1216 respondents, who are individuals living in Vietnam. It can be seen that the authors have addressed the research questions by pointing out that energy-saving policy does not significantly influence the energy-saving intentions of households. However, other factors such as attitudes, subjective norms, energy prices, perceived usefulness, and the impact of the COVID-19 crisis on the energy-saving intentions and behaviors of households. In particular, this research also highlights that the PMT plays an important role in explaining energy-saving intentions and behaviors in the context of crises like COVID-19. Households tend to be more inclined to save in the face of social upheavals or economic uncertainty. Based on the findings of this research, the authors have identified several implications.

### 5.2 Theoretical implications

The study makes a significant contribution to explaining the suitability of the TPB and the TAM in explaining energy-saving behavior in households. It is evident that the TAM model is a good fit for explaining energy-saving behavior in households. Factors such as the ease of using energy-saving devices, the perceived usefulness of such devices, subjective norms, and attitudes can all explain the intentions and behaviors related to energy-saving in households. On the other hand, the TPB model does not fully explain the energy-saving intentions of households, as the behavior control factor is not related to energy-saving intentions. Additionally, the study's significant contribution lies in uncovering the impact of the COVID-19 crisis on energy-saving intentions and behaviors. The results demonstrate that the PMT can explain the effects of crises on energy-saving behaviors in households. Moreover, the SET is also supported in this research, indicating that energy-saving policies play a role in household energy-saving decisions.

### 5.3 Policy implications

This study will support policy makers to develop appropriate strategies to improve energy saving attitudes, intentions and behaviors of households in particular as well as households in general. First, the findings underscore the importance of promoting energy-saving appliances as a means to enhance household energy efficiency. Research indicates that consumers exhibit a positive attitude towards energy-efficient appliances, which significantly influences their purchasing intentions. This suggests that policymakers should prioritize the development and promotion of user-friendly, energy-efficient products. By implementing policies that incentivize the production and adoption of such appliances, households may be more inclined to adopt energy-saving behaviors, thereby contributing to overall energy conservation efforts.

Moreover, raising awareness of social responsibility and social norms can play a critical role in shaping energy-saving intentions. Therefore, policies aimed at enhancing public awareness of energy conservation, coupled with campaigns that emphasize collective responsibility, can foster a culture of energy efficiency. This aligns with findings that suggest a strong correlation between social norms and energy-saving behaviors, indicating that individuals are more likely to engage in energy-saving practices when they perceive such behaviors as socially endorsed.

Additionally, the implementation of tiered energy pricing can serve as an effective mechanism to encourage energy-saving behaviors among households. Research supports the notion that economic incentives, such as differentiated pricing based on consumption levels, can significantly influence households' energy-saving intentions. By structuring energy pricing to reward lower consumption, policymakers can create a financial motivation for households to adopt energy-efficient practices, thus facilitating a shift towards more sustainable energy usage.

Furthermore, the integration of environmental education into community programs can enhance energy-saving awareness and behaviors. Evidence suggests that educational initiatives that increase knowledge about energy conservation can lead to improved attitudes and intentions towards energy-saving actions. Therefore, policymakers should consider developing comprehensive educational campaigns that not only inform households about the benefits of energy conservation but also provide practical guidance on implementing energy-saving measures in daily life.

## 6 Limitation and future research

Despite research indicating the impact of energy prices, energy-saving policies, and crises on energy-saving behavior, there remain several limitations within the studies conducted. Firstly, the sampling method employed was convenience sampling, which may not accurately reflect the overall population in terms of gender, regional distribution, or other demographic characteristics. Secondly, the research was conducted in the post-COVID-19 period, suggesting that the influence of crises on energy-saving behavior could still be significant. It remains unclear whether this impact will change over time, necessitating further investigation.

From these limitations, the study proposes several recommendations for future research on similar topics. Firstly, subsequent studies could aim to collect data from multiple provinces with a sample ratio that closely aligns with the overall population demographics. Secondly, longitudinal studies could provide deeper insights into how perceptions and behaviors related to crises and energy-saving practices evolve over time. Such research could enhance our understanding of the dynamics between crisis situations and energy-saving behaviors, ultimately contributing to more effective energy conservation strategies.

## CRediT authorship contribution statement

**Dat Ngoc Nguyen:** Writing – review & editing, Writing – original draft, Supervision, Resources, Project administration, Methodology, Funding acquisition. **Duy Van Nguyen:** Writing – original draft, Validation, Software, Resources, Methodology, Data curation. **Dat Dinh Nguyen:** Investigation, Formal analysis, Data curation, Conceptualization. **Thuy Trong Than:** Writing – original draft, Visualization, Software, Investigation. **Loc Xuan Tran:** Writing – review & editing, Methodology, Investigation.

## Ethics approval and consent to participate

This study was initiated under Degree No. 052022/QD-QAglobal issued by the Quantitative Analysis Center, QAglobal, Vietnam. QAglobal is Duy Van Nguyen's old institution (Corresponding author) before moving to Phenikaa University. Respondents agreed to participate in the study after being fully informed, and respondents participated in the survey voluntarily.

## Data and code availability

Data will be made available on request. For requesting data, please write to the corresponding author.

## Funding

This research is funded by Foreign Trade University under research program number FTURP02-2023-12.

## Declaration of competing interest

The authors declare that they have no known competing financial interests or personal relationships that could have appeared to influence the work reported in this paper.
