# Supplementary material for: How do energy price, energy-saving policies, and crisis affect energy-saving behavior?
Source: Heliyon. 2025 Feb 19;11(4):e42787. doi: 10.1016/j.heliyon.2025.e42787 (PMC11904500; doi:10.1016/j.heliyon.2025.e42787)
Supplement: Multimedia component 1 [file mmc1.docx]

**QUESTIONNAIRE**

**Part 1. Please tick your level of agreement with the statements stated in the questions below. in which the:**

1- Strongly disagree, 2- Disagree, 3- Normal, 4- Agree; 5- Strongly agree

| **Code** | | **Content** | | **Level** | | | | | | | |  |
| --- | --- | --- | --- | --- | --- | --- | --- | --- | --- | --- | --- | --- |
| ***I. Subject Norm*** | | | | | | | | | | | |  |
| SNO1 | Households need to be conscious of energy-saving behavior | | | 1 | 2 | | 3 | | 4 | | 5 |  |
| SNO2 | You are influenced by the people around you (family, friends, or neighbors) | | | 1 | 2 | | 3 | | 4 | | 5 |  |
| SNO3 | Actively participate in electricity saving activities when society has many popular electricity saving activities. | | | 1 | 2 | | 3 | | 4 | | 5 |  |
| ***II. COVID19*** | | | | | | | | | | | |  |
| COVID1 | | COVID-19 reduces your income | | 1 | | 2 | | 3 | | 4 | 5 |  |
| COVID2 | | COVID-19 changes your work | | 1 | | 2 | | 3 | | 4 | 5 |  |
| COVID3 | | In general, you are greatly affected by COVID-19 | | 1 | | 2 | | 3 | | 4 | 5 |  |
| ***III.* Policy** | | | | | | | | | | | |  |
| PO1 | You think the energy saving policy is effective | | | 1 | 2 | | 3 | | 4 | | 5 |  |
| PO2 | You think that energy saving policies benefit everyone | | | 1 | 2 | | 3 | | 4 | | 5 |  |
| ***IV. Perceived easy of use*** | | | | | | | | | | | |  |
| PEU1 | You think the Energy Saving Device is easy to operate | | | 1 | 2 | | 3 | | 4 | | 5 |  |
| PEU2 | You think that Energy Saving Appliances are easy to repair | | | 1 | 2 | | 3 | | 4 | | 5 |  |
| ***VI. Price*** | | | | | | | | | | | |  |
| PRI1 | You think energy prices are high compared to your income | | | 1 | 2 | | 3 | | 4 | | 5 |  |
| PRI2 | According to you, electricity prices tend to increase every year | | | 1 | 2 | | 3 | | 4 | | 5 |  |
| PRI3 | Electricity prices are your concern | | | 1 | 2 | | 3 | | 4 | | 5 |  |
| ***VI. Perceived of usefulness*** | | | | | | | | | | | |  |
| PU1 | Using energy-efficient equipment helps you save costs. | | | 1 | 2 | | 3 | | 4 | | 5 |  |
| PU2 | Using energy-saving equipment helps protect the environment | | | 1 | 2 | | 3 | | 4 | | 5 |  |
| ***VII. Behavior control*** | | | | | | | | | | | |  |
| CON1 | You understand energy saving skills | | | 1 | 2 | | 3 | | 4 | | 5 |  |
| CON2 | Implementing energy savings is easy | | | 1 | 2 | | 3 | | 4 | | 5 |  |
| CON3 | You're always in control of your energy savings | | | 1 | 2 | | 3 | | 4 | | 5 |  |
| ***VIII. Attitude*** | | | | | | | | | | | |  |
| ATT1 | You think saving energy is necessary | | | 1 | 2 | | 3 | | 4 | | 5 |  |
| ATT2 | You think that saving energy is protecting the living environment in the long term | | | 1 | 2 | | 3 | | 4 | | 5 |  |
| ﻿ATT3 | You think that saving energy helps reduce energy consumption for daily activities | | | 1 | 2 | | 3 | | 4 | | 5 |  |
| ***IX. Intention*** | | | | | | | | | | | |  |
| INT1 | You always plan to save energy | | |  |  | |  | |  | |  |  |
| INT2 | You will buy energy efficient appliances when possible | | |  |  | |  | |  | |  |  |
| INT3 | You will remind others when possible | | |  |  | |  | |  | |  |  |
| ***X. Behavior*** | | | | | | | | | | | |  |
| BE1 | Always turn off electrical equipment when not in use | | | 1 | 2 | | 3 | | 4 | | 5 |  |
| BE2 | You always use energy-efficient equipment | | | 1 | 2 | | 3 | | 4 | | 5 |  |
| BE3 | You always use reminding others about saving energy | | | 1 | 2 | | 3 | | 4 | | 5 |  |
| **Part 2: Personal Information**  Please provide information by ticking the corresponding box below: | | | | | | | | | | | | |
| 1. Gender   ❒ Male | | | ❒ Female | | | | | | | | | |
| 1. Education   ❒ High School ❒ Colleage ❒ Graduate University ❒ Master/PhD | | | | | | | | | | | | |
| 1. Occupation   ❒ Offical staff ❒ Unemployment ❒ Worker ❒ Engineer  ❒ Self-employed        ❒ Lecturer/teacher. ❒ Others | | | | | | | | | | | | |
| 1. Income   ❒ under 10 million VND/month ❒ 10- under 15 million VND/month  ❒ 15- under 20 million VND/month. ❒ >20million VND/month | | | | | | | | | | | | |
|  | | | | | | | | | | | | |

**THANK YOU SO MUCH!**
