# Supplementary material for: How do energy price, energy-saving policies, and crisis affect energy-saving behavior?
Source: Heliyon. 2025 Feb 19;11(4):e42787. doi: 10.1016/j.heliyon.2025.e42787 (PMC11904500; doi:10.1016/j.heliyon.2025.e42787)
Supplement: Multimedia component 2 [file mmc2.docx]

Apply for Article Publication waiver for manuscript: “**How do energy price, energy-saving policies, and crisis affect energy-saving behaviour?”.**

Dear Editor officer,

We would like to apply a waiver of the article publishing charges (APC) for our manuscript with the following details:

**Manuscript title: “How do energy price, energy-saving policies, and crisis affect energy-saving behaviour”**

**First Author:** Dat Ngoc Nguyen

**Institution:** Foreign Trade University

**Address**: 91 Chua Lang st, Hanoi, Vietnam

**Other authors:** Vietnam

**Reason for applying for a waiver:** We are researchers from Vietnam, a resource-scare setting. This research results from a domestic scientific network for research capacity building in Vietnam with very limited funding received, and we have mostly spent for conducting the research. Thus, it is challenging for us to manage budget to cover USD 2100 of APC. It would be great if you have any special policy for encouraging researchers from low-middle-income countries like Vietnam (based on World Bank list of economies). In addition, we hope that this manuscript will be global interested if it is published in Heliyon. Therefore, we strongly appreciate your consideration and any support to waive the APC.

Thank you very much,

Sincerely
